# The association of travel distance and other patient characteristics with breast cancer stage at diagnosis and treatment completion at a rural Rwandan cancer facility

**DOI:** 10.1186/s12885-025-13489-2

**Published:** 2025-01-27

**Authors:** Kayleigh Bhangdia, Abirami Natarajan, Niclas Rudolfson, Stéphane Verguet, Marcia C. Castro, Jean-Marie Vianney Dusengimana, Cyprien Shyirambere, Lauren E. Schleimer, Lawrence N. Shulman, Aline Umwizerwa, Catherine Kigonya, John Butonzi, Emily MacDuffie, Temidayo Fadelu, Daniel S. O’Neil, Cam Nguyen, Tharcisse Mpunga, Nancy L. Keating, Lydia E. Pace

**Affiliations:** 1 Institute for Health Metrics and Evaluation, Seattle, WA USA; 2 Dana-Farber Cancer Institute, Boston, MA USA; 3 Lund University, Lund, Sweden; 4 Department of Global Health and Population, Harvard T. H. Chan School of Public Health, Boston, MA USA; 5 Partners in Health Rwanda/Inshuti Mu Buzima, Butaro, Rwanda; 6 Department of Surgery, Columbia University Medical Center, New York, NY USA; 7 Abramson Cancer Center, University of Pennsylvania, Philadelphia, PA USA; 8 Ministry of Health, Kigali, Rwanda; 9 Department of Internal Medicine & Yale Cancer Center, Yale University School of Medicine, New Haven, CT USA; 10 University of Colorado Anschutz Medical Campus, Aurora, CO USA; 11 University Teaching Hospital of Kigali, Kigali, Rwanda; 12 Brigham and Women’s Hospital, 75 Francis Street, Boston, MA 02115 USA; 13 Harvard Medical School, Boston, MA USA

**Keywords:** Breast cancer, Distance, Travel, Global cancer, Rwanda, Africa, Disparities, Outcomes, Cancer stage

## Abstract

**Background:**

Butaro Cancer Center of Excellence (BCCOE) was founded to serve Rwanda’s rural low-income population, providing subsidized cancer diagnosis and treatment with transport stipends for the lowest-income patients. We examined whether travel distance to BCCOE was associated with advanced-stage diagnoses and treatment completion.

**Methods:**

We conducted a retrospective cohort study using medical record data from BCCOE patients with pathologically-confirmed breast cancer from 2012–2016. Women with no prior surgery were included in the stage analysis; those with non-metastatic disease were included in the treatment analysis. We calculated travel distances using spatial analytic software and used multivariable logistic regression to examine the association of distance and other patient characteristics with late-stage diagnoses and treatment completion within one year of diagnosis.

**Results:**

The analytic cohort for stage included 426 patients; 75.1% had late-stage (stage 3 or 4) disease. In univariable analyses, patients residing in BCCOE’s surrounding district had a lower proportion of late-stage diagnoses compared to those residing outside the district (57.9% v 76.8%, *p* = 0.02). In adjusted analyses, odds of late-stage diagnosis were 2.46 (95% CI:1.21–5.12) times higher among those in distance quartile 4 (> 135.8 km) versus 1 (< 55.7 km); the effect of distance was less strong in sensitivity analyses excluding patients from BCCOE’s surrounding district. Patients from sectors with > 50% poverty had 2.33 times higher odds of late-stage diagnoses (95% CI:1.07–5.26) relative to those with poverty < 30%. In the treatment completion cohort (*n* = 348), 49.1% of patients completed surgery and chemotherapy within a year. In adjusted analyses, travel distance and poverty were not linearly associated with treatment completion.

**Conclusions:**

At Rwanda’s first public cancer facility, sector-level poverty and longer travel distances were associated with late-stage breast cancer diagnoses, but less clearly associated with treatment completion, perhaps partly due to travel stipends provided to the lowest-income individuals undergoing treatment. Our findings support further investigation into wider use of travel stipends to facilitate early diagnosis and treatment completion.

**Supplementary Information:**

The online version contains supplementary material available at 10.1186/s12885-025-13489-2.

## Background

Breast cancer incidence and mortality are rapidly increasing in sub-Saharan Africa. Though the region’s breast cancer incidence remains lower than high-income countries’, women in sub-Saharan Africa face a greater likelihood of dying from breast cancer [1–3]. The African Breast Cancer-Disparities in Outcomes (ABC-DO) prospective cohort study of women diagnosed with breast cancer in 5 sub-Saharan African countries showed a 3-year crude survival of 50% [4], compared with 85–90% in high-income countries [5]. The ABC-DO study also illuminated substantial inequities in stage at diagnosis, treatment completion, and survival both between and within sub-Saharan African countries [4, 6]. Understanding strategies to reduce these inequities and optimize breast cancer outcomes in the region is a global public health priority [7].

Patients in sub-Saharan Africa often must travel long distances to receive specialty cancer care, contributing to high direct and indirect costs borne by patients for cancer diagnosis and treatment. [8] Globally, longer travel distance to cancer facilities has been associated with later-stage diagnoses [9, 10], lower rates of treatment completion [8] and worse survival [10]. Providing travel stipends and decentralizing cancer diagnostics and treatment have been recommended as strategies to reduce the burden and expense of travel for cancer care and improve outcomes [4, 6].

Rwanda is a low-income East African country of 10,169 square miles [11] with roughly 13 million inhabitants [12], most of whom are subsistence farmers. In 2012, Rwanda’s Ministry of Health, in collaboration with the non-governmental organization Partners In Health (PIH) and the Dana-Farber Cancer Institute, launched Butaro Cancer Center of Excellence (BCCOE), Rwanda’s first comprehensive cancer care facility. BCCOE was opened within Butaro District Hospital in Burera District, a rural, mountainous district in Rwanda’s Northern Province with a low-income population. Through its partnership with PIH, BCCOE provides highly subsidized cancer services, minimizing patients’ medical costs. Additionally, BCCOE’s lowest-income patients receive transport stipends for travel to and from the hospital after their initial visit [13, 14]. Though cancer care has expanded to other facilities in Rwanda, services at BCCOE remain the most affordable and the most commonly utilized among Rwanda’s rural poor. Thus, while BCCOE’s rural location brought cancer care to a previously underserved region of the country, the facility also serves as a cancer referral center for the entire country [15].

BCCOE has provided a model for cancer care delivery in a rural sub-Saharan African setting. However, patients seeking care at BCCOE still face challenges, particularly those associated travel. BCCOE’s rural, northwestern location can be hard to reach for patients coming from other equally underserved regions of the country. Furthermore, patients are only eligible for socioeconomic support after establishing care at BCCOE, so care subsidies and transport stipends do not ease barriers to pre-diagnostic and diagnostic services. Finally, patients whose incomes are above eligibility thresholds for transport sipends often still face financial difficulties associated with travel; in one survey of 89 women receiving treatment for breast cancer at BCCOE, the median reported cost of round-trip travel to the hospital was 12 USD for a single visit, about 75% of a Rwandan adult’s average monthly consumption [16]. To explore how these factors may impact equitable breast cancer care, we sought to examine the relationship between patients’ travel distance to BCCOE and stage at breast cancer diagnosis as well as the association of distance with completion of treatment within one year.

## Methods

### Data sources

Patient data for this analysis were collected from BCCOE electronic and paper medical records using structured extraction forms. We abstracted age at presentation; year of presentation; medical comorbidities; prior treatment; stage at diagnosis; hormone receptor status; treatment course; and residential village location. For this analysis, we included treatment data for up to a year after patients’ diagnosis.

All data used for the geographical analysis were downloaded from open source websites. Rwandan subnational administrative boundary shapefiles were from the World Health Organization’s Humanitarian Data Exchange database developed for the 2012 Census and updated in 2018 [17]. A road shapefile was downloaded from OpenStreetMap (2020), and a digital elevation model (DEM, of 90 m) was used from the ArcGIS hub (2018) [18, 19]. The land use and landcover dataset with associated speed limits was obtained from Google Earth Engine [20], and road speeds were taken from Munoz et al. [21].

Data on poverty rates came from Rwanda’s 2013–2014 Poverty Mapping Report [22], which provides area-level rates of poverty as defined by Rwanda’s 2013–2014 Poverty Profile Report [23]. Researchers in Rwanda’s National Institute of Statistics conducted a detailed survey of 14,419 households to determine rates of poverty as measured by per-adult consumption, for example purchases of food, non-food goods, and services such as education [24]. Poverty was defined as consumption below a level required to enable basic nutritional requirements and essential non-food requirements (i.e. Rwanda’s poverty line; 159,375 Rwandan francs per adult per year in January 2014, or about 239 USD.). To create poverty maps, researchers developed a predictive model for poverty and applied this to the Rwanda 2012 Housing Census to estimate cell- and sector-level poverty rates for every region of Rwanda. In our analysis we chose to use poverty rates by sector, the third level of administrative sub-division in Rwanda. Rwanda has 416 sectors [25].

### Study population

**Fig. 1:**
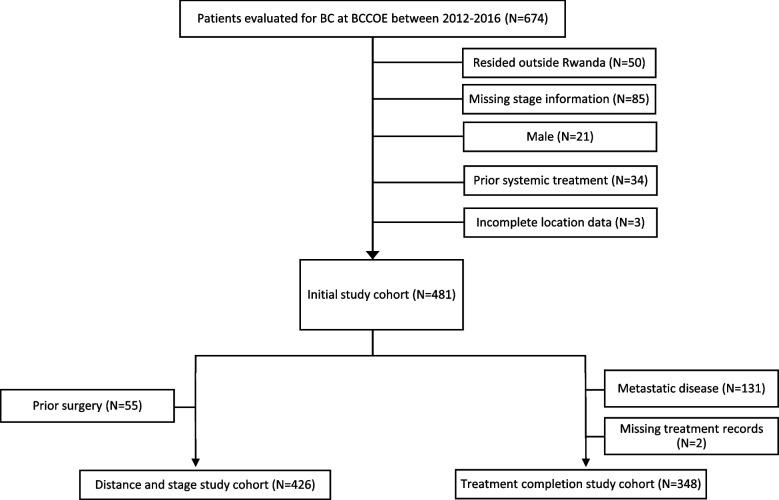
Cohort-building process

The study population included female patients with pathologically-confirmed breast cancer who were evaluated at BCCOE between 2012–2016. Patients were excluded if there was incomplete data on their residence, if they resided outside of Rwanda, were missing data needed to determine stage at diagnosis, or had received any systemic treatment for breast cancer before their first visit at BCCOE. For the stage analysis, we additionally excluded patients who had received surgery at a different facility before their evaluation at BCCOE, since we were interested in assessing factors associated with stage upon initial presentation at BCCOE. For the treatment completion analysis, we included patients who had had surgery prior to BCCOE presentation, but excluded patients with stage IV disease who were not eligible for curative treatment and patients for whom treatment records could not be located (Fig. 1). For both the staging and treatment analyses, we included patients who had surgery outside of BCCOE after their first presentation at BCCOE, since when a surgeon was not available at BCCOE, BCCOE clinicians often arranged for patients to receive surgery at other facilities.

### Key variables

#### Outcome variables

1. Stage at diagnosis. At BCCOE, breast cancer is staged according to the American Joint Committee on Cancer (AJCC) version 7 anatomic staging system [26]. When stage was not explicitly stated in a patient’s medical record, researchers abstracting medical records determined a patient’s stage based on documented tumor size at presentation, the presence of palpable lymph nodes on physical exam, and the results of imaging (typically chest x-ray and liver ultrasound). When all information needed to determine clinical stage was not available in medical records, we used surgical pathology reports to determine pathologic stage among those who had mastectomies (but no neoadjuvant therapy). All staging determinations made by researchers were reviewed and approved by BCCOE clinicians. For the stage analysis, cases were categorized as either early (Stage 0, 1, or 2) or late stage (Stage 3 or 4).
2. Treatment completion. Treatment completion was defined based on protocols in use at BCCOE during the study period and BCCOE-specific breast cancer treatment quality measures developed through a Delphi panel of Rwanda-based and global experts [27]. For all patients, complete treatment included undergoing surgery within one year of diagnosis. Receipt of at least four cycles of chemotherapy (e.g. four cycles of doxorubicin/cyclophosphamide) within one year of diagnosis was also required for all patients with hormone receptor (HR) negative cancer and for all patients with regional lymph node involvement either on physical examination or based on surgical pathology. We excluded endocrine therapy from our definition of treatment completion so as to not bias our results against patients with HR positive cancer. Of note, *HER2*-targeted therapy was not available during the study period, and *HER2* testing was infrequently performed, so the chemotherapy regimen provided was doxorubicin/cyclophosphamide typically followed by paclitaxel, in line with National Comprehensive Cancer Network Harmonized Guidelines for Sub-Saharan Africa [28]. Radiotherapy was not available in Rwanda during the time of this study. While in rare situations, patients obtained radiotherapy privately outside of Rwanda, few patients with breast cancer at BCCOE received radiotherapy.

#### Independent variables

1. Travel distance. After uploading and preparing our data in AccessMod5, the accessibility analysis tool was utilized to calculate the most efficient travel distance from each patient’s home to BCCOE based on landcover, road networks, and travel speeds associated with those roads (which impacted selection of the most efficient route). We also calculated the travel distance from each patient to their nearest health center using the referral analysis tool.
2. Residential sector poverty level. Using data from Rwanda’s 2013–2014 Poverty Mapping Report [22], each patient was assigned a relative poverty index based on their sector of residence, representing the percentage of households in the sector that were living below the poverty line [22]; this was categorized as < 30%, 30–50%, and > 50% poverty.
3. Other covariates included age, the presence of any comorbidities as identified in a pre-specified list on patients’ clinical intake form (including HIV, hypertension, diabetes, hepatitis, heart failure and others), HR status, and year of diagnosis.

### Statistical analysis

1. Stage. Chi-square tests were used to examine unadjusted associations between patient travel distance quartile, other characteristics, and late stage presentations. A multivariable logistic model was used to adjust for all covariates described above and included in Table 1.

**Table 1:** Characteristics of patients included in analysis of breast cancer stage at diagnosis at Butaro Cancer Center of Excellence, 2012–2016

	**All Patients**	**Late Stage N(%)**	*p*-value*
N	**426**	**320 (75.1)**	
**Year of presentation**			
2012	62	54 (87.1)	0.12
2013	110	84 (76.4)	
2014	94	70 (74.5)	
2015	95	65 (68.4)	
2016	65	47 (72.3)	
**Age group (years)**			
< 40	109	79 (72.5)	0.65
40–60	234	176 (75.2)	
> 60	83	65 (78.3)	
**Households living in poverty in sector of residence**			
< 30%	112	78 (69.6)	0.26
30—50%	237	181 (76.4)	
> 50%	77	61 (79.2)	
**Distance quartiles to BCCOE (km)**			
Quartile 1 (< = 55.7)	107	74 (69.2)	0.07
Quartile 2 (55.8–90.2)	106	79 (74.5)	
Quartile 3 (92.3–135.8)	106	77 (72.6)	
Quartile 4 (> 135.8)	107	90 (84.1)	
**Residence in Burera district**			
No	388	298 (76.8)	**0.02**
Yes	38	22 (57.9)	
**Distance quartiles to health center (km)**			
Quartile 1 (< = 1.68)	108	77 (71.3)	0.53
Quartile 2 (1.68–2.97)	107	84 (78.5)	
Quartile 3 (2.98–5.05)	104	81 (77.9)	
Quartile 4 (> 5.05)	107	78 (72.9)	
**Comorbidities**			
No	311	233 (74.9)	0.98
Yes	115	87 (75.7)	
**Hormone receptor positive**			
No	144	121 (84)	**0.01**
Yes	262	186 (71)	
Unknown	20	13 (65)	

^*^Univariable analyses using Chi-square tests comparing the proportion of individuals with early versus late-stage disease, by patient characteristic
2. Treatment Completion. Chi-square tests were used to examine unadjusted associations between patient travel distance, other characteristics, and treatment completion. We constructed a multivariable logistic regression model including all available covariates that clinical experience and existing literature suggested might be associated with likelihood of treatment completion.
3. Sensitivity analyses. We performed 2 sensitivity analyses. First, we were aware that, despite high poverty rates (Appendix Fig. 1), patients residing in Burera District, the district immediately surrounding BCCOE, faced several advantages regarding early breast cancer diagnosis beyond shorter travel distances. One advantage was that during the study period, those living in Burera could be referred from their health center directly to BCCOE’s specialty oncology clinic rather than having to go to a district hospital general clinic first. In contrast, in order for national health insurance to cover medical services, those residing outside of Burera had to be referred from health centers to their own district’s hospital, and then to BCCOE [29]. A second advantage was that in 2015, a breast cancer early detection intervention was implemented at 12 randomly selected health centers in Burera district [30]. This intervention consisted of training community health workers in breast awareness, and health center nurses on clinical breast assessment [30, 31]. Since this intervention increased diagnoses of early-stage cancer, we identified whether patients had been exposed to the early detection intervention based on their residential area and timing of their first presentation at BCCOE. To explore whether living in Burera offered potential advantages in addition to shorter travel distances, we conducted a sensitivity analysis in which we included a separate distance category for Burera patients and other Quartile 1 patients.

Second, we noted that among our treatment completion cohort, almost 2/3 of mastectomies received were performed outside of BCCOE, since during some of our study period there was not a surgeon on-site. Thus, we performed a second sensitivity analysis in which we examined only completion of at least 4 cycles of chemotherapy as our treatment completion outcome, to investigate whether this outcome was more sensitive to travel distance and other patient characteristics.

All analyses were conducted using R (version 4.0.0) and SAS (version 9.4) statistical software.

## Results

### Cohort

Four-hundred twenty-six patients were included in the stage analysis; 348 were included in the treatment completion analysis (Fig. 1).

### Stage at diagnosis

**Fig. 2:**
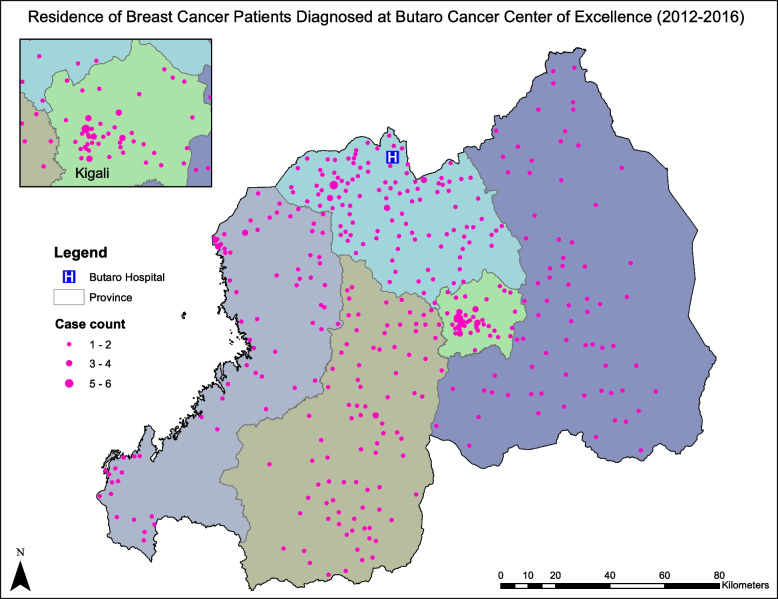
Residence of breast cancer patients diagnosed at Butaro Cancer Center of Excellence (2012–2016)

Table 1 shows characteristics of the stage analysis cohort and results of univariate analysis. Of the 426 patients included in the stage analysis, 75.1% (*N* = 320) were diagnosed with late-stage (stage 3 or 4) disease. Median age at first visit to BCCOE was 49 years (interquartile range (IQR): 40–58 years). Median travel distance to patients’ local health centers was 3.0 km; median travel distance to BCCOE was 90.1 km. The distribution of patients’ residences relative to BCCOE is shown in Fig. 2. About three-quarters lived in geographic sectors with a poverty rate of 30% or more. In univariate analyses, 69.2% of patients living within the first distance quartile (≤ 55.7 km) from BCCOE had late-stage disease at diagnosis, versus 74.5% in quartile 2, 72.6% in quartile 3, and 84.1% in quartile 4 (*p* = 0.07). Among patients living in Burera District (*n* = 38), 57.9% of cancer diagnoses were late-stage (*p* = 0.02). When we examined the subset of 12 Burera patients referred from health centers that participated in the 2015–2017 early detection intervention, 6 (50%) had late-stage diagnoses (*p* = 0.09) (data not shown). Patients living in sectors with > 50% poverty rates had higher rate of late-stage diagnoses (69.2%, 74.5%, 84.1% among those from sectors with < 30%, 30–50%, and > 50% poverty, respectively) though this difference was not statistically significant (*p* = 0.07). Patients with HR-negative disease (*n* = 144), were also more likely to have late-stage disease (84% among those with HR-negative disease versus 71% among those with HR-positive disease and 65% among those with unknown HR status, *p* = 0.01).

Table 2 shows results from the multivariable logistic regression model. The adjusted odds of late stage diagnosis were 2.46 (95% CI: 1.22–5.12) times higher among those in distance quartile 4 compared to those in quartile 1. Those residing in sectors with > 50% poverty had 2.33 (95% CI: 1.07–5.26) times greater odds of late-stage diagnosis. Later years of diagnosis were associated with lower likelihood of late-stage diagnosis compared to 2012, and HR positivity was associated with lower likelihood of late stage diagnosis versus negative or unknown HR status. In the sensitivity analysis that included a category for Burera patients separately from distance quartiles 1–4, we found that patients from outside of Burera District but still in distance quartile 1, had 3.68 times greater odds of late-stage diagnoses relative to the 38 Burera patients.(Appendix Table 1) Patients living in distance quartile 4 also had greater odds of late-stage diagnoses. In a model excluding Burera patients, distance quartiles were no longer statistically significantly associated with late-stage diagnoses, though there was a trend towards a greater odds of late-stage diagnoses among patients in distance quartile 4 patients compared to those in quartile 1 (adjusted OR (aOR) 1.76, 95% CI: 0.80–4.02) (Appendix Table 2).

**Table 2:** Multivariable logistic regression model to examine factors associated with late stage at breast cancer diagnosis (*n* = 426)

Variable	Adjusted Odds Ratio^a^	95% CI
**Year of presentation**		
2012	Reference	-
2013	0.51	(0.20—1.19)
2014	**0.40**	**(0.15—0.95)**
2015	**0.30**	**(0.12—0.71)**
2016	**0.38**	**(0.14 – 0.96)**
**Age group (years)**		
< 40	Reference	-
40—60	1.09	(0.63—1.86)
> 60	1.25	(0.62- 2.54)
**Households living in poverty in sector of residence**		
< 30%)	**Reference**	-
30–50%	1.55	(0.90—2.67)
> 50%	**2.33**	**(1.07—5.26)**
**Distance quartiles to BCCOE (km)**		
Quartile 1 (< = 55.7)	Reference	-
Quartile 2 (55.8–90.2)	1.42	(0.72—2.84)
Quartile 3 (92.3–135.8)	1.29	(0.65—2.52)
Quartile 4 (> 135.8)	**2.46**	**(1.21 – 5.12)**
**Comorbidities**		
None	Reference	-
One or more	0.96	(0.57—1.64)
**Hormone receptor positive**		
No	Reference	-
Yes	**0.52**	**(0.31—0.85)**

^a﻿^Adjusting for all variables in the table

### Treatment completion

Treatment completion analysis results are in Table 3. Of 348 patients with nonmetastatic disease, 172 (49.4%) completed surgery and chemotherapy when recommended within one year. In unadjusted analyses, treatment completion rates differed by distance quartile, with those living in the closest and furthest quartiles having the highest rates of treatment completion (55.7% and 56.8%, respectively, versus 35.6% and 49.4% for quartiles 2 and 3; *p* = 0.02). Younger age was associated with treatment completion, with women aged 40–60 years having the highest rates of treatment completion (56.9%), compared to only 30.0% of women over 60 years (*p* < 0.001). Those diagnosed in 2012 and 2013 (the first 2 years of the program) had lower rates of treatment completion (38.8% and 39.6%, respectively) than in 2014 (55.6%), 2015 (48.2%) and 2016 (70.6%) (*p* = 0.003). Sector-level poverty rate (< 30%, 30–50%, > 50%) was not associated with treatment completion in univariate analyses. In adjusted analyses, travel distance in quartile 2 versus quartile 1 was associated with lower adjusted odds of treatment completion (aOR 0.36, 95% CI: 0.17–0.75), as was age > 60 relative to patients aged 40–60 years (aOR 0.33, 95% CI: 0.18–0.61). Those residing in sectors in the middle range of poverty rates (30–50% living in poverty) were less likely to complete treatment than those who lived in the wealthiest sectors (aOR 0.55, 95% CI: 0.31–0.98), but there was no difference for those living in the highest-poverty sectors. In the sensitivity analysis examining completion of 4 cycles of chemotherapy only, findings were overall similar; however in multivariable regression distance and poverty were not associated with odds of chemotherapy completion (Appendix Table 3). Women with later-stage or hormone receptor negative cancer were more likely to complete chemotherapy, likely because it was emphasized more in those patients.

**Table 3:** Unadjusted and adjusted associations between patient characteristics and treatment completion within a year after diagnosis, among patients with non-metastatic breast cancer (*n* = 348)

Variable	Completed treatment N (%)	*p*-value*	Adjusted OR (95% CI)^a^
**Overall**	172 (49.4)		
**Distance quartile to BCCOE (km)**			
Quartile 1 (< 56.2)	49 (55.7)	**0.02**	Ref
Quartile 2 (56.2–90.6)	31 (35.6)		**0.36 (0.17, 0.75)**
Quartile 3 (90.7–142.2)	42 (49.4)		0.61 (0.30, 1.22)
Quartile 4 (> 142.2)	50 (56.8)		0.96 (0.48, 1.91)
**Age group** (years)		** < 0.001**	
< 40	36 (47.4)		0.67 (0.38, 1.20)
40—60	115 (56.9)		Ref
> 60	21 (30.0)		**0.33 (0.18, 0.61)**
**Year of presentation**			
2012	17 (38.6)	**0.003**	Ref
2013	38 (39.6)		1.07 (0.49, 2.34)
2014	40 (55.6)		1.81 (0.79, 4.13)
2015	41(48.2)		1.38 (0.62, 3.06)
2016	36 (70.6)		**2.64 (1.06, 6.61)**
**Hormone receptor status**		0.11	
Positive	94 (45.9)		0.68 (0.42, 1.10)
Negative or unknown	78 (54.6)		Ref
**Stage**		0.52	
I/II	62 (52.1)		1.18 (0.72, 1.91)
III	110 (48.0)		Ref
**Percent of households in poverty at sector level**			
< 30%	52 (53.1)	0.65	Ref
30–50%	93 (47.5)		**0.54 (0.30, 0.96)**
> 50%	27 (50.0)		0.57 (0.25, 1.27)
**Comorbidities**			
Yes	42 (44.7)	0.28	0.75 (0.45, 1.27)
No	130 (51.2)		Ref

*OR* Odds Ratio

^*^Univariable analyses using Chi-square tests comparing the proportion of individuals who completed versus did not complete treatment within a year, by characteristic

^a^Multivariable logistic regression, adjusting for all variables in the table

## Discussion

This analysis of breast cancer stage at presentation and treatment completion in the first cancer care facility in Rwanda demonstrates both challenges and opportunities for equitable breast cancer diagnosis and care provision in a resource-constrained rural environment.

We found that living in a high-poverty sector was an important predictor of late-stage diagnoses in Rwanda, similar to other settings in sub-Saharan Africa. While travel distance to BCCOE appeared to play a role in late-stage diagnoses, factors beyond simply distance, such as the presence of a local early detection intervention and more streamlined referral pathways, also likely contributed to the higher rates of earlier-stage diagnoses among patients in the hospital’s surrounding areas. In contrast, among women with non-metastatic breast cancer, distance and poverty were not consistently associated with treatment completion.

Late-stage diagnoses were prevalent in our cohort, similar to elsewhere in sub-Saharan Africa. Similar to other studies [8], patients’ distance to their local primary care facilities did not affect breast cancer stage at diagnosis, likely because most Rwandans live close to their local health center. However, also consistent with other studies, even in the relatively small country of Rwanda, those with the longest travel distances (over 135.8 km) to the cancer diagnosis and treatment facility had later stage at diagnoses than those living close by. This finding was less marked when patients from the hospitals immediately surrounding district were excluded likely partly due to the smaller sample size as well as the contribution of other factors described below. The role of distance in impeding early-stage diagnoses supports findings from interviews with Rwandan clinicians and patients who described travel distances to referral facilities as a major barrier to timely breast cancer diagnosis [32, 33]. Decentralizing diagnostic services or providing transport support during the pre-diagnostic phase of patients’ journeys could mitigate some of these barriers. Updated analyses are also needed to examine the impact of growing community awareness and access on stage at presentation.

In addition to patient factors, systems factors also influenced early stage presentation in this cohort. Patients from BCCOE’s surrounding Burera district had lower rates of late-stage diagnoses even compared with patients who lived equally close to the hospital but in other districts. This may reflect in part more efficient referral processes experienced by patients living in the same district as BCCOE. This pattern was also seen in South Africa, where patients who were required to have a secondary-level hospital visit prior to breast cancer diagnosis at a tertiary hospital had 50% higher likelihood of late-stage diagnosis [29]. The Burera district early detection intervention also likely contributed to higher rates of early-stage diagnoses in Burera [30]. These findings underscore the potential role of expedited referral pathways in early cancer diagnosis and the importance of building clinicians’ and facilities’ capacity to rapidly identify and refer those with concerning breast findings.

Our study also found that living in higher-poverty regions was associated with later stage at presentation, even after adjusting for distance. Barriers faced by lower-income patients could include the cost of insurance (although Rwanda’s insurance premiums are free for the lowest-income patients, they can be a barrier for those who don’t meet those criteria), indirect costs of cancer treatment such as transportation, and competing priorities, stigma, and low health literacy, including the perception that cancer cannot be treated. These poverty-related barriers are supported by survey and qualitative data we have previously gathered in Rwanda [32–34].

In contrast to stage at diagnosis, among patients with non-metastatic disease, the relationship between distance and treatment completion at BCCOE was less straightforward. We observed that patients with the shortest and longest distances had higher odds of treatment completion than those living in the two middle distance quartiles. This finding may have several explanations. The subset of patients whose disease was diagnosed at BCCOE before it had progressed to stage 4, despite living far away, may have been advantaged in unmeasurable ways and *more* likely to be able to adhere to treatment. Additionally, it is possible that BCCOE’s travel subsidies for patients undergoing treatment may reduce the burden of travel for some patients, mitigating the role of distance. Only the lowest-income patients (defined based on household poverty classifications established by the government used across Rwanda) [35] qualify for these subsidies, and patients who do not meet criteria may still struggle to cover travel costs; this could explain our finding that patients from areas with the highest rates of poverty were no less likely to complete treatment compared with patients from the wealthiest areas, whereas patients from areas with mid-range poverty rates were less likely. The nuanced relationship between poverty, distance, and treatment completion may suggest that enhanced support for patients (together with BCCOE’s location in a rural, low-income location) may be helping the most vulnerable obtain treatment. Expanded eligibility for financial support could improve treatment completion overall. However, further research is needed to explore this.

Our study has several strengths. To our knowledge, it is one of the first studies of breast cancer in sub-Saharan Africa that uses a rasterized distance analysis that takes into account road networks, elevation, landcover and travel speeds to identify the most efficient routes that better reflect the reality for patients, as opposed to Euclidean straight-line distance calculations that are more prone to inaccurate estimates. It is also the first to examine contributors to breast cancer treatment completion in Rwanda after initiation of Rwanda’s innovative approach to cancer care in a rural underserved area.

Our study has several limitations. First, because we relied on routine medical record data, we lacked information on important covariates including detailed patient-level socioeconomic information and which patients received transport stipends. We used sector-level poverty, which has not been validated as a strategy to estimate individual Rwandans’ socioeconomic position. Second, BCCOE patients may not be representative of all patients with breast cancer throughout Rwanda. For example, those who could afford private health services in Kigali or outside the country might prefer to receive care and treatment elsewhere. Finally, our data reflect the first cohort of patients cared for at BCCOE; updated analyses will be important to examine how stage and treatment completion have evolved among Rwandans with breast cancer seen at BCCOE.

## Conclusion

A growing body of evidence demonstrates the barriers facing women in Sub-Saharan Africa who require high-quality, timely breast cancer diagnosis and treatment. Our study’s findings underscore the potential value of decentralizing diagnostic services and social/ financial support for patients to reduce the detrimental effects of travel distance on timely diagnosis, while also suggesting the importance of health system interventions to streamline referrals and build clinician capacity in early diagnosis. Further study is urgently needed to investigate the impact of services such as transport stipends in facilitating treatment completion.

## Supplementary Information

Supplementary Material 1.

Supplementary Material 2.

Supplementary Material 3.

Supplementary Material 4.

Supplementary Material 5.

Supplementary Material 6.

## Data Availability

No datasets were generated or analysed during the current study.

## Abbreviations

ABC-DO: African Breast Cancer-Disparities in Outcomes
PIH: Partners In Health
BCCOE: Butaro Cancer Center of Excellence
AJCC: American Joint Committee on Cancer
HR: Hormone receptor
IQR: Interquartile range

## References

[1] Gakwaya A, Kigula-Mugambe JB, Kavuma A, Luwaga A, Fualal J, Jombwe J, et al. Cancer of the breast: 5-year survival in a tertiary hospital in Uganda. Br J Cancer. 2008;99(1):63–7. 18577991 10.1038/sj.bjc.6604435 PMC2453032

[2] Pace LE, Dusengimana J-MV, Hategekimana V, Habineza H, Bigirimana JB, Tapela N, et al. Benign and Malignant Breast Disease at Rwanda’s First Public Cancer Referral Center. Oncologist. 2016;21(5):571–5. 27009935 10.1634/theoncologist.2015-0388 PMC4861361

[3] Pace LE, Shulman LN. Breast Cancer in Sub-Saharan Africa: Challenges and Opportunities to Reduce Mortality. Oncologist. 2016;21(6):739–44. 27091419 10.1634/theoncologist.2015-0429 PMC4912363

[4] McCormack V, McKenzie F, Foerster M, Zietsman A, Galukande M, Adisa C, et al. Breast cancer survival and survival gap apportionment in sub-Saharan Africa (ABC-DO): a prospective cohort study. Lancet Glob Health. 2020;8(9):e1203–12. 32827482 10.1016/S2214-109X(20)30261-8 PMC7450275

[5] Allemani C, Matsuda T, Di Carlo V, Harewood R, Matz M, Nikšić M, et al. Global surveillance of trends in cancer survival 2000–14 (CONCORD-3): analysis of individual records for 37 513 025 patients diagnosed with one of 18 cancers from 322 population-based registries in 71 countries. Lancet. 2018;391(10125):1023–75. 29395269 10.1016/S0140-6736(17)33326-3 PMC5879496

[6] Foerster M, McCormack V, Anderson BO, Boucheron P, Zietsman A, Cubasch H, et al. Treatment guideline concordance, initiation, and abandonment in patients with non-metastatic breast cancer from the African Breast Cancer-Disparities in Outcomes (ABC-DO) cohort in sub-Saharan Africa: a prospective cohort study. Lancet Oncol. 2022;23(6):729–38. 35550274 10.1016/S1470-2045(22)00198-X PMC10036870

[7] Anderson BO, Ilbawi AM, Fidarova E, Weiderpass E, Stevens L, Abdel-Wahab M, et al. The Global Breast Cancer Initiative: a strategic collaboration to strengthen health care for non-communicable diseases. Lancet Oncol. 2021;22(5):578–81. 10.1016/S1470-2045(21)00071-1. Epub 2021 Mar 7 10.1016/S1470-2045(21)00071-1 33691141

[8] Togawa K, Anderson BO, Foerster M, Galukande M, Zietsman A, Pontac J, et al. Geospatial barriers to healthcare access for breast cancer diagnosis in sub-Saharan African settings: The African Breast Cancer—Disparities in Outcomes Cohort Study. Int J Cancer. 2021;148(9):2212–26. 33197280 10.1002/ijc.33400 PMC8048597

[9] Dickens C, Joffe M, Jacobson J, Venter F, Schüz J, Cubasch H, et al. Stage at breast cancer diagnosis and distance from diagnostic hospital in a periurban setting: a South African public hospital case series of over 1,000 women. Int J Cancer. 2014;135(9):2173–82. 24658866 10.1002/ijc.28861 PMC4134722

[10] Knapp GC, Tansley G, Olasehinde O, Wuraola F, Adisa A, Arowolo O, et al. Geospatial access predicts cancer stage at presentation and outcomes for patients with breast cancer in southwest Nigeria: A population-based study. Cancer. 2021;127(9):1432–8. 33370458 10.1002/cncr.33394 PMC8404086

[11] University of Pennsylvania. Rwanda - Geography. East Africa Living Encyclopedia. https://www.africa.upenn.edu/NEH/rwgeography.htm/. Accessed 2 June 2023.

[12] World Bank Group. Population, total - Rwanda. World Bank Open Data; 2022. https://data.worldbank.org/indicator/SP.POP.TOTL?locations=RW/. Accessed 28 Aug 2023.

[13] Shulman LN, Mpunga T, Tapela N, Wagner CM, Fadelu T, Binagwaho A. Bringing cancer care to the poor: experiences from Rwanda. Nat Rev Cancer. 2014;14(12):815–21. 25355378 10.1038/nrc3848

[14] Stulac S, Binagwaho A, Tapela NM, Wagner CM, Muhimpundu MA, Ngabo F, et al. Capacity building for oncology programmes in sub-Saharan Africa: the Rwanda experience. Lancet Oncol. 2015;16(8):e405–13. 26248848 10.1016/S1470-2045(15)00161-8

[15] Tapela NM, Mpunga T, Hedt-Gauthier B, Moore M, Mpanumusingo E, Xu MJ, Nzayisenga I, et al. Pursuing equity in cancer care: implementation, challenges and preliminary findings of a public cancer referral center in rural Rwanda. BMC Cancer. 2016;16:237.10.1186/s12885-016-2256-7. 10.1186/s12885-016-2256-7 PMC4797361 26992690

[16] Fadelu T, Nguyen C, Nsabimana N, Bigirimana E, Mukandayisenga V, Tuyishime V, et al. Abstract 67: Quantifying Transportation Barriers in Rwandan Patients Seeking Treatment for Breast Cancer. Cancer Epidemiol Biomarkers Prev. 2021;3(7):67. 10.1158/1538-7755.Asgcr21-67.

[17] United Nations Office for the Coordination of Humanitarian Affairs. Rwanda - Subnational Administrative Boundaries Humanitarian Data Exchange. The Humanitarian Data Exchange. https://data.humdata.org/dataset/cod-ab-rwa/. Accessed 3 Feb 2023.

[18] ArcGIS. Open Street Map data for Rwanda. https://www.arcgis.com/home/item.html?id=aa3f9b10f98844a088f1ba393b818648/. Accessed 3 Feb 2023.

[19] ArcGIS. Rwanda 90 meters Digital Elevation Model. https://hub.arcgis.com/datasets/dbe91ec91b7740bdbab44e29d2c954db/. Accessed 3 Feb 2023.

[20] Google. Google Earth Engine; 2021. https://earthengine.google.com/. Accessed 3 Feb 2023.

[21] Huerta Munoz U, Källestål C. Geographical accessibility and spatial coverage modeling of the primary health care network in the Western Province of Rwanda. Int J Health Geogr. 2012;11(1):40. 22984920 10.1186/1476-072X-11-40 PMC3517388

[22] National Institute of Statistics Rwanda. Poverty Mapping Report 2013–2014. The Republic of Rwanda; 2017. https://www.statistics.gov.rw/publication/poverty-mapping-report-2013-2014/. Accessed 3 Feb 2023.

[23] National Institute of Statistics Rwanda. Rwanda Poverty Profile Report - Results of EICV 4. The Republic of Rwanda; 2015. https://www.statistics.gov.rw/publication/rwanda-poverty-profile-report-results-eicv-4/. Accessed 28 Aug 2023.

[24] Deaton, A. Zaidi, S. Guidelines for constructing consumption aggregates for welfare analysis (English). Living standards measurement study (LSMS) working paper, no. LSM 135. World Bank Group; 2002. https://documents.worldbank.org/en/publication/documents-reports/documentdetail/206561468781153320/guidelines-for-constructing-consumption-aggregates-for-welfare-analysis/.

[25] Ministry of Local Government. Administrative Structure Rwanda: Government of Rwanda; 2023. Available from: https://www.gov.rw/government/administrative-structure.

[26] Amin MB, Greene FL, Edge SB, Compton CC, Gershenwald JE, Brookland RK, et al. The Eighth Edition AJCC Cancer Staging Manual: Continuing to build a bridge from a population-based to a more "personalized" approach to cancer staging. CA Cancer J Clin. 2017;67(2):93–9. 10.3322/caac.21388. Epub 2017 Jan 17 10.3322/caac.21388 28094848

[27] Pace LE, Schleimer LE, Shyirambere C, Ilbawi A, Dusengimana JMV, Bigirimana JB, et al. Identifying Breast Cancer Care Quality Measures for a Cancer Facility in Rural Sub-Saharan Africa: Results of a Systematic Literature Review and Modified Delphi Process. JCO Glob Oncol. 2020;6:1446–54. 32997538 10.1200/GO.20.00186 PMC7529520

[28] National Comprehensive Cancer Network. NCCN Harmonized Guidelines for Sub-Saharan Africa. NCCN Guidelines and Clinical Resources. 2017. Available from: https://www.nccn.org/harmonized.

[29] Mapanga W, Norris SA, Craig A, Ayeni OA, Chen WC, Jacobson JS, et al. Drivers of disparities in stage at diagnosis among women with breast cancer: South African breast cancers and HIV outcomes cohort. PLoS ONE. 2023;18(2): e0281916. 36795733 10.1371/journal.pone.0281916 PMC9934316

[30] Pace LE, Dusengimana JMV, Shulman LN, Schleimer LE, Shyirambere C, Rusangwa C, et al. Cluster Randomized Trial to Facilitate Breast Cancer Early Diagnosis in a Rural District of Rwanda. Journal of Global Oncology. 2019;5:1–13. 31774713 10.1200/JGO.19.00209 PMC6882507

[31] Pace LE, Dusengimana JMV, Keating NL, Hategekimana V, Rugema V, Bigirimana JB, et al. Impact of Breast Cancer Early Detection Training on Rwandan Health Workers’ Knowledge and Skills. Journal of Global Oncology. 2018;4:1–10. 30241228 10.1200/JGO.17.00098 PMC6223427

[32] Uwimana A, Dessalegn S, Vianney Dusengimana JM, Stauber C, Fata A, Hagenimana M, et a. Integrating Breast Cancer Early Detection Into a Resource-Constrained Primary Health Care System: Health Care Workers' Experiences in Rwanda. JCO Glob Oncol. 2022;8:e2200181. 10.1200/GO.22.00181. 10.1200/GO.22.00181 PMC10166372 36508703

[33] Pace LE, Fata AM, Cubaka VK, Nsemgiyumva T, Uwihaye JD, Stauber C, et al. Patients' experiences undergoing breast evaluation in Rwanda's Women's Cancer Early Detection Program. Breast Cancer Res Treat. 2023;202(3):541–50. 10.1007/s10549-023-07076-x. Epub 2023 Aug 30 10.1007/s10549-023-07076-x PMC11670140 37646967

[34] Pace LE, Mpunga T, Hategekimana V, Dusengimana JM, Habineza H, Bigirimana JB, et al. Delays in Breast Cancer Presentation and Diagnosis at Two Rural Cancer Referral Centers in Rwanda. Oncologist. 2015;20(7):780–8. 26032138 10.1634/theoncologist.2014-0493 PMC4492236

[35] Local Administrative Entities Development Agency (LODA). Cabinet approves review and classification of households into Ubudehe categories2020. Available from: https://loda.prod.risa.rw/updates/news-detail/cabinet-approves-review-and-classification-of-households-into-ubudehe-categories#:~:text=After%20reintroduction%20in%202001%2C%20the,%2C%20and%20(6)%20Abakire. Cited 2024 September 10.

