# Supplementary material for: The association of travel distance and other patient characteristics with breast cancer stage at diagnosis and treatment completion at a rural Rwandan cancer facility
Source: BMC Cancer. 2025 Jan 27;25:146. doi: 10.1186/s12885-025-13489-2 (PMC11771020; doi:10.1186/s12885-025-13489-2)
Supplement: Supplementary file 1 — Supplementary Material 1. [file 12885_2025_13489_MOESM1_ESM.pdf]

Appendix Figure 1. Sector-level poverty versus travel distance to Butaro Center of Excellence among patients diagnosed with breast cancer

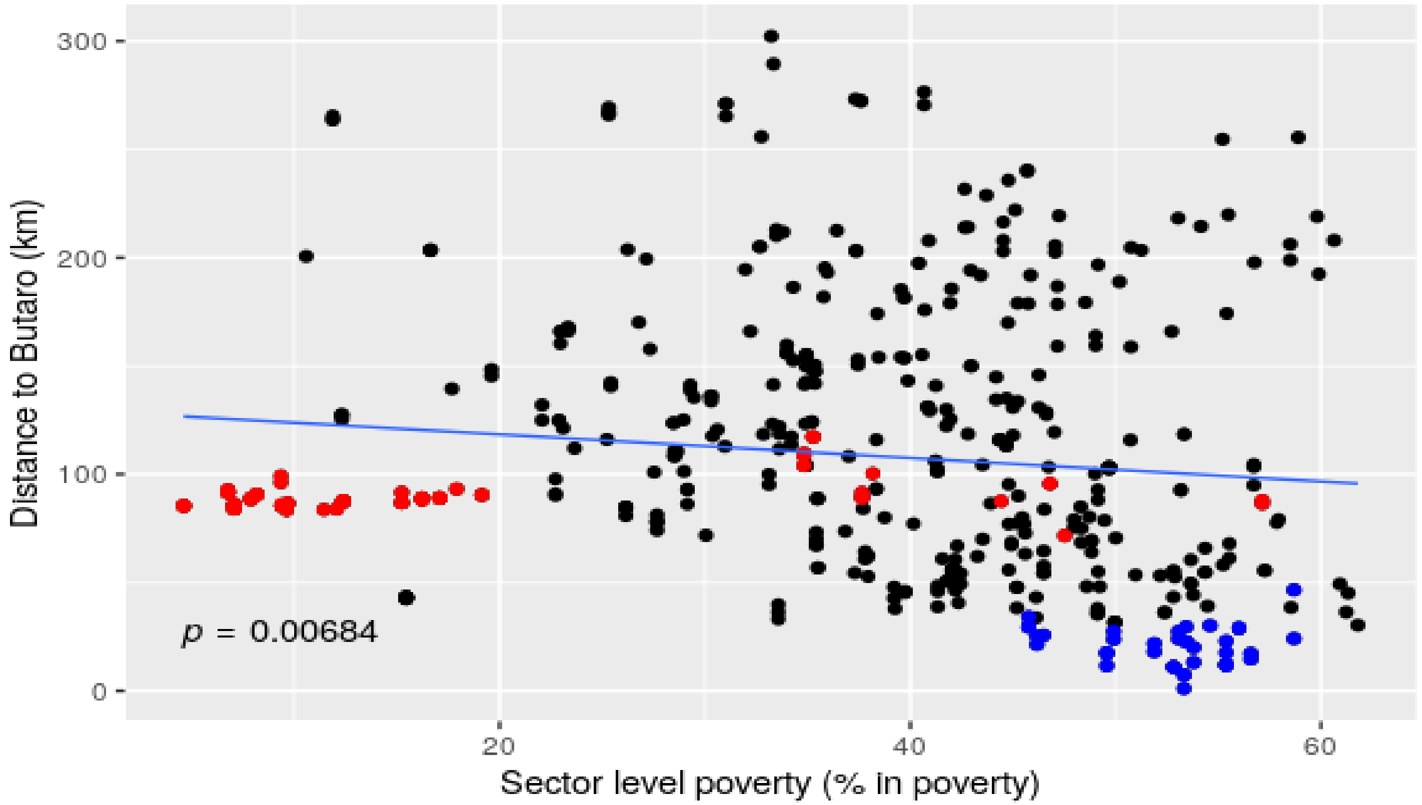


Red = Kigali province; blue = Burera District
