# Supplementary figures and images for: The association of travel distance and other patient characteristics with breast cancer stage at diagnosis and treatment completion at a rural Rwandan cancer facility
Source: BMC Cancer. 2025 Jan 27;25:146. doi: 10.1186/s12885-025-13489-2 (PMC11771020; doi:10.1186/s12885-025-13489-2)

Appendix Figure 2. Percent of patients with breast cancer diagnosed at stage 3 or 4, 2012-2016

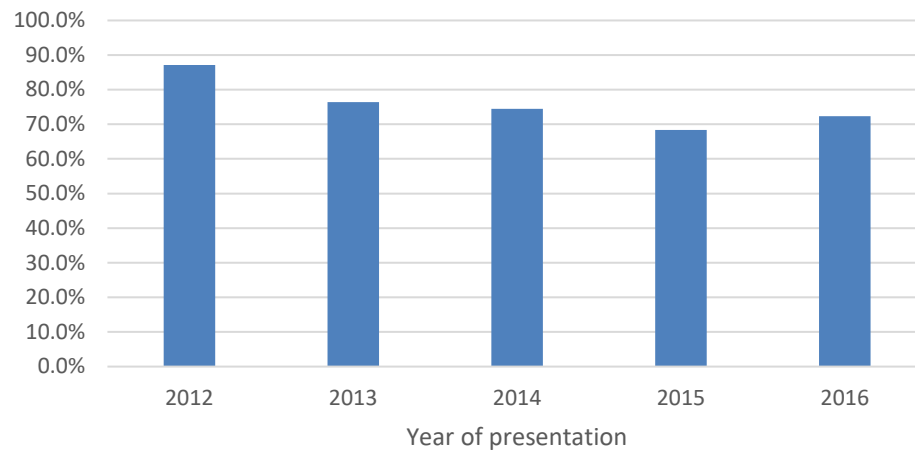

Supplement: Supplementary file 2 — Supplementary Material 2. [file 12885_2025_13489_MOESM2_ESM.pdf]
