# Supplementary material for: The association of travel distance and other patient characteristics with breast cancer stage at diagnosis and treatment completion at a rural Rwandan cancer facility
Source: BMC Cancer. 2025 Jan 27;25:146. doi: 10.1186/s12885-025-13489-2 (PMC11771020; doi:10.1186/s12885-025-13489-2)
Supplement: Supplementary file 3 — Supplementary Material 3. [file 12885_2025_13489_MOESM3_ESM.pdf]

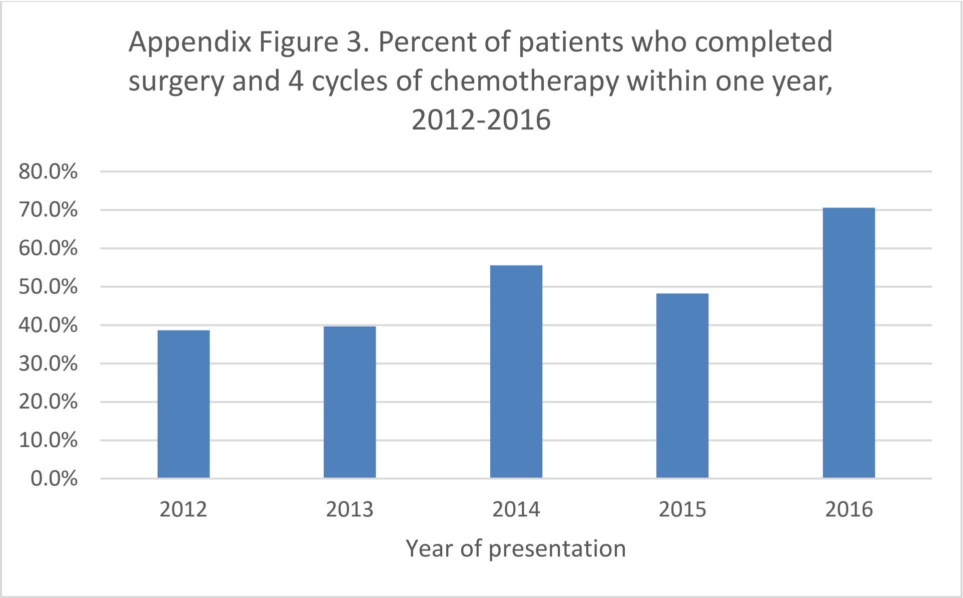
Appendix Figure 3. Percent of patients who completed surgery and 4 cycles of chemotherapy within one year, 2012-2016
80.0%
70.0%
60.0%
50.0%
40.0%
30.0%
20.0%
10.0%
0.0%
2012
2013
2014
2015
2016
Year of presentation
