# Supplementary material for: The association of travel distance and other patient characteristics with breast cancer stage at diagnosis and treatment completion at a rural Rwandan cancer facility
Source: BMC Cancer. 2025 Jan 27;25:146. doi: 10.1186/s12885-025-13489-2 (PMC11771020; doi:10.1186/s12885-025-13489-2)
Supplement: Supplementary file 4 — Supplementary Material 4. [file 12885_2025_13489_MOESM4_ESM.docx]

| Appendix Table 1. Multivariable logistic regression model to examine factors associated with late stage at breast cancer diagnosis, with Burera District residence examined separately from distance quartiles (n=426) | | |
| --- | --- | --- |
| **Variable** | **Adjusted Odds Ratio*** | **95% CI** |
| **Distance to BCCOE** (km) |  |  |
| Burera district | Reference | - |
| Quartile 1 (non-Burera) | **3.68** | **(1.45 - 9.62)** |
| Quartile 2 (non-Burera) | 2.33 | (0.83- 6.72) |
| Quartile 3 (non-Burera) | **2.76** | **(1.02 - 7.70)** |
| Quartile 4 (non-Burera) | **6.22** | **(2.21 - 18.30)** |
| **Age group (years)** |  |  |
| < 40 | Reference | - |
| 40 - 60 | 1.13 | (0.65 - 1.95) |
| > 60 | 1.34 | (0.65- 2.80) |
| **Year** |  |  |
| 2012 | Reference | - |
| 2013 | 0.53 | (0.21 - 1.25) |
| 2014 | **0.37** | **(0.14 - 0.91)** |
| 2015 | **0.30** | **(0.12 - 0.72)** |
| 2016 | 0.43 | (0.15 - 1.11) |
| **Percent of households in poverty at sector level** |  |  |
| Category 1 (<30%) | Reference | - |
| Category 2 (30-50%) | 1.39 | (0.77 - 2.49) |
| Category 3 (>50%) | **2.77** | **(1.14 - 7.17)** |
| **Comorbidities** | 0.90 | (0.53 - 1.56) |
| **Health center participation in early detection intervention** | 0.45 | (0.12 - 1.79) |
| **Hormone receptor positive** | **0.52** | **(0.31 - 0.85)** |

*Adjusted for all variables in the table.
