# Supplementary material for: The association of travel distance and other patient characteristics with breast cancer stage at diagnosis and treatment completion at a rural Rwandan cancer facility
Source: BMC Cancer. 2025 Jan 27;25:146. doi: 10.1186/s12885-025-13489-2 (PMC11771020; doi:10.1186/s12885-025-13489-2)
Supplement: Supplementary file 5 — Supplementary Material 5. [file 12885_2025_13489_MOESM5_ESM.docx]

| Appendix Table 2. Multivariable logistic regression model to examine factors associated with late stage at breast cancer diagnosis, with Burera District patients excluded (n= 388) | | |
| --- | --- | --- |
| **Variable** | **OR** | **95% CI** |
| **Distance to BCCOE** (km) |  |  |
| Quartile 1 (<=55.7) | Reference | - |
| Quartile 2 (55.8-90.2) | 0.66 | (0.30- 1.42) |
| Quartile 3 (92.3-135.8) | 0.80 | (0.38 - 1.64) |
| Quartile 4 (>135.8) | 1.76 | (0.80 - 4.02) |
| **Age group (years)** |  |  |
| < 40 | Reference | - |
| 40 - 60 | 1.04 | (0.57 - 1.84) |
| > 60 | 1.42 | (0.65- 3.25) |
| **Year** |  |  |
| 2012 | Reference | - |
| 2013 | 0.51 | (0.19 - 1.26) |
| 2014 | **0.36** | **(0.13 - 0.94)** |
| 2015 | **0.27** | **(0.10 - 0.66)** |
| 2016 | **0.34** | **(0.11 - 0.93)** |
| **Percent of households in poverty at sector level** |  |  |
| Category 1 (<30%) | Reference | - |
| Category 2 (30-50%) | 1.33 | (0.74 - 2.41) |
| Category 3 (>50%) | **3.83** | **(1.36 - 12.86)** |
| **Comorbidities** | 0.94 | (0.53 - 1.68) |
| **Health center participation in early detection intervention** | 0.47 | (0.08 - 3.74) |
| **Hormone receptor positive** | **0.48** | **(0.27 - 0.81)** |
