# Supplementary material for: The association of travel distance and other patient characteristics with breast cancer stage at diagnosis and treatment completion at a rural Rwandan cancer facility
Source: BMC Cancer. 2025 Jan 27;25:146. doi: 10.1186/s12885-025-13489-2 (PMC11771020; doi:10.1186/s12885-025-13489-2)
Supplement: Supplementary file 6 — Supplementary Material 6. [file 12885_2025_13489_MOESM6_ESM.docx]

Appendix Table 3. Unadjusted and adjusted associations between patient characteristics and completion of 4 cycles of chemotherapy within a year after diagnosis, among patients with non-metastatic breast cancer among whom chemotherapy was indicated (n=334)

| **Variable** | **Completed 4 cycles of chemotherapy**  **N (%)** | **p-value*** | **Adjusted OR (95% CI)**** |
| --- | --- | --- | --- |
| **Overall** | 205 (61.4) |  |  |
| **Distance quartile to BCCOE (km)** |  |  |  |
| Quartile 1 (<56.9) | 54 (63.5) | 0.18 | Ref |
| Quartile 2 (56.9-91.2) | 42 (51.2) |  | 0.50 (0.23, 1.08) |
| Quartile 3 (91.3-147.5) | 53 (63.9) |  | 0.75 (0.36, 1.58) |
| Quartile 4 (>147.5) | 56 (66.7) |  | 0.94 (0.45, 1.96) |
| **Age group** (years) |  | **<0.001** |  |
| < 40 | 43 (58.1) |  | 0.64 (0.35, 1.16) |
| 40 - 60 | 133 (69.6) |  | Ref |
| > 60 | 29 (42.0) |  | **0.31 (0.17, 0.57)** |
| **Year of presentation** |  |  |  |
| 2012 | 21 (47.7) | **<0.001** | Ref |
| 2013 | 49 (53.3) |  | 1.34 (0.63, 2.87) |
| 2014 | 43 (64.2) |  | 1.97 (0.86, 4.51) |
| 2015 | 51 (60.7) |  | 1.74 (0.80, 3.83) |
| 2016 | 41 (87.2) |  | **2.64 (1.06, 6.61)** |
| **Hormone receptor status** |  | **0.02** |  |
| Positive | 107 (56.0) |  | 0.65 (0.39, 1.07) |
| Negative or unknown | 98 (68.5) |  | Ref |
| **Stage** |  | 0.52 |  |
| I/II | 57 (54.3) |  | 0.59 (0.35, 0.99**)** |
| III | 148 (64.6) |  | Ref |
| **Percent of households in poverty at sector level** |  |  |  |
| <30% | 58 (63.0) | 0.88 | Ref |
| 30-50% | 117 (61.3) |  | 0.69 (0.37, 1.26) |
| >50% | 30 (58.8) |  | 0.60 (0.26, 1.41) |
| **Comorbidities** |  |  |  |
| Yes | 52 (57.8) | 0.41 | 0.81 (0.47, 1.39) |
| No | 153 (62.7) |  | Ref |

OR = Odds Ratio. *Univariable analyses using Chi-square tests comparing the proportion of individuals who completed versus did not complete treatment within a year, by characteristic; **Multivariable logistic regression
